# Physical Characterization to Improve Scalability and Potential of Anesthetic-Loaded Nanodroplets

**DOI:** 10.3390/pharmaceutics15082077

**Published:** 2023-08-03

**Authors:** Siulam Ginni Ting, Harriet Lea-Banks, Kullervo Hynynen

**Affiliations:** 1 Physical Sciences Platform, Sunnybrook Research Institute, Toronto, ON M4N 3M5, Canada; harriet.lea-banks@sri.utoronto.ca; 2 Department of Medical Biophysics, University of Toronto, Toronto, ON M5S 1A1, Canada; 3 Institute of Biomedical Imaging, University of Toronto, Toronto, ON M5S 1A1, Canada

**Keywords:** focused ultrasound, nanodroplets, targeted drug delivery, acoustics, drug release

## Abstract

Drug-loaded perfluorocarbon nanodroplets (NDs) can be activated non-invasively by focused ultrasound (FUS) and allow for precise drug-delivery. Anesthetic-loaded NDs and transcranial FUS have previously achieved targeted neuromodulation. To assess the clinical potential of anesthetic-loaded NDs, in depth physical characterization and investigation of storage strategies and triggered-activation is necessary. Pentobarbital-loaded decafluorobutane nanodroplets (PBNDs) with a Definity-derived lipid shell (237 nm; 4.08 × 10^9^ particles/mL) were fabricated and assessed. Change in droplet stability, concentration, and drug-release efficacy were tested for PBNDs frozen at −80 °C over 4 weeks. PBND diameter and the polydispersity index of thawed droplets remained consistent up to 14 days frozen. Cryo-TEM images revealed NDs begin to lose circularity at 7 days, and by 14 days, perfluorocarbon dissolution and lipid fragmentation occurred. The level of acoustic response and drug release decreases through prolonged storage. PBNDs showed no hemolytic activity at clinically relevant concentrations and conditions. At increasing sonication pressures, liquid PBNDs vaporized into gas microbubbles, and acoustic activity at the second harmonic frequency (2 f_0_) peaked at lower pressures than the subharmonic frequency (1/2 f_0_). Definity-based PBNDs have been thoroughly characterized, cryo-TEM has been shown to be suitable to image the internal structure of volatile NDs, and PBNDs can be reliably stored at −80 °C for future use up to 7 days without significant degradation, loss of acoustic response, or reduction in ultrasound-triggered drug release.

## 1. Introduction

As of 2022, there are around 100 nanomedicines with regulatory approval worldwide [1], up from 51 approved nanomedicines in 2013 [2]. The field of nanomedicine has enjoyed significant advances due to scientific breakthroughs in nanoscale materials science, biomedical science, and pharmaceutical science [3]. In particular, lipid-based drug delivery systems have received attention due to their relatively easy manufacturing process, biocompatibility, capability for temporally or spatially controlled drug release, and compatibility with both hydrophilic and hydrophobic drugs [4]. Clinical need has also been a key driver, illustrated by the rapid design, approval, and manufacturing of lipid-based nanoparticles for vaccine delivery seen during the COVID-19 (coronavirus 2019) pandemic [5]. Focused ultrasound (FUS) is a method to achieve triggered drug release in superheated nanodroplets (NDs) by vaporizing the liquid core, thereby releasing the pre-loaded pharmaceutical in the intended localized area [6].

Since the 1970s, the United States Food and Drug Administration (FDA) received over 600 applications, which include investigational new drugs (IND, determine eligibility to begin clinical trial based on safety profile), new drug applications (NDA, to gain approval for marketing), and abbreviated new drug applications (ANDA, submissions for generic versions of already approved drugs). A study found that for nanomedicine, 19% of INDs resulted in a submitted NDA, 15% of NDAs were approved, and 43% of ANDAs were approved; numbers that were higher than average across all drug products [7]. One possible reason for this relatively high success rate is due to researchers building off pre-existing formulations and systems that have already been approved or have well-investigated safety profiles. Although there has been a shift to favor investigation of increasingly complex nanoparticle drug delivery systems, ~70% of the submissions to the FDA involve “first-generation” nanoparticles, such as liposomes, nanocrystals, and iron colloids [7].

Despite the benefits of lipid-based drug delivery systems, technical and regulatory challenges hinder clinical translation. A better understanding of key roadblocks or potential barriers could improve the development of lipid-based nanotherapeutics to expedite the journey from conception to marketed production. One technical aspect related to production is storage and stability. The ability to be premade and stored for extended periods of time is extremely advantageous for nanocarriers, but prolonged storage in cold conditions of lipid-based nanoparticles can yield the possibility of drug expulsion from an encapsulated form due to crystallization and other polymorphic changes in the lipid structures [4]. A thorough investigation into both the optimal storage conditions and maximum storage time without compromising therapeutic efficacy could hasten a nanoparticle’s path to clinical translation. Considerations of technical factors could improve the end product’s usage and effectiveness by accounting for healthcare environmental settings.

A nanoparticle’s storage capabilities can also impact the pharmacoeconomic aspects of clinical translation. When making decisions on drug programs and reimbursement guidelines, a thorough analysis of a drug’s clinical effectiveness, the economic impact (including direct and indirect costs which contribute to economic burden), and the impact on the healthcare system is conducted [8,9]. The enhanced storage capabilities of lipid-based nanoparticles will bring about significant pharmacoeconomical advantages and improve the cost-effectiveness of the drug delivery system. Extended storage can reduce the frequency of restocking, reduce the preparation time for usage, and ultimately substantially reduce costs for healthcare providers and patients alike. An examination of the storage capabilities of nanomedicines could pave the way for more affordable and accessible healthcare solutions, with the end goal of improving patient outcomes while efficiently managing healthcare expenditures [10].

Prior to regulatory approval, thorough characterization to ensure the physicochemical properties of drug nanocarriers can be reliably reproduced is paramount. Examples of physicochemical characterization commonly include particle diameter, particle size distribution, morphology, and drug release profiles [11]. As lipid-based nanoparticles such as NDs become more complex with various surface customizations, lipid formulations, liquid cores, and loaded drugs [12,13], the need to confirm that the final product contains all the necessary components in the correct ratios becomes increasingly important. Absorbance, fluorescence, mass spectrometry, and thin layer chromatography are examples of methods for determining concentrations of the desired components and allow for quantification of drug loading or other desired moieties in in the nanoparticle [14]. Surface charge and electrostatic potential (zeta potential) can affect molecular interactions in vivo, which can lead events such as cell wall disruptions, or preferential binding to certain inter/intracellular structures. Laser Doppler velocimetry is a method by which zeta potential can be obtained [14].

Past studies involving low boiling point perfluorocarbon NDs have used the above-listed methods to fully characterize their drug-loaded nanodroplets, namely through delineation of the NDs’ size distribution, drug loading, drug released (spontaneous and FUS-triggered), and zeta potential [6,15,16,17,18,19].

In the field of nanomedicine, one of the most common causes of clinical trial termination or discontinuation from the market and failure to achieve regulatory approval is nanomedicine toxicity [20]. Some parameters to investigate that would mitigate clinical translation obstacles stem from immunology (e.g., in vivo immune response), hematology (e.g., hemolysis assay), in vivo single dose toxicity studies, and biodistribution studies. For studies aiming to ascertain the safety of their nanoparticle prior to in vivo experiments, a hemolysis assay or a cytotoxicity assay to measure the amount of lysis in erythrocytes or seeded cells in vitro as a result of incubation with the tested nanotherapeutic is sufficient.

Here, we thoroughly perform physical characterization of pentobarbital-loaded, lipid-shell, decafluorobutane nanodroplets (PBNDs) for non-invasive neuromodulation, and show that short-term frozen storage is a feasible and convenient solution for clinical use without compromising efficacy. We report the morphology, internal structure, sizing, concentration, suitability for prolonged frozen storage at −80 °C, spontaneous drug release, droplet activity in vitro, and potential for hemolysis of PBNDs.

## 2. Materials and Methods

### 2.1. Materials

Decafluorobutane (DFB, C_4_F_10_) was purchased from Synquest Labs (Alachua, FL, USA), pentobarbital solution from Sigma Aldrich (Millipore Sigma; St. Louis, MO, USA), and Definity from Lantheus Medical Imaging, USA. In its commercially available form, Definity has a lipid shell composed of (R)-4-hydroxy-N,N,N-trimethyl-10-oxo-7-[(1oxohex-adecyl)oxy]-3,4,9-trioxa-4-phosphapentacosan-1-aminium, 4-oxide (DPPC); (R)-hexadecanoic acid, 1-[(phosphonoxy)methyl]-1,2-ethane-diyl ester (DPPA); and (R)-∝-[6-hydroxy-6-oxido-9-[(1-oxohexadecyl) oxy]-5,7,11-trioxa-2aza-6-phosphahexacos-1-yl]-ω-methoxypoly(ox-1,2-ethanediyl) (MPEG5000 DPPE), at a mole percentage ratio of 82:10:8, giving a lipid concentration of 0.75 mg/mL, which encapsulates a gaseous octofluoropropane (OFP, C_3_F_8_) core. Milli-Q ultrapure water (Millipore Sigma, USA) was used throughout the fabrication.

### 2.2. Droplet Fabrication

Pentobarbital-loaded nanodroplets (PBNDs) with a final drug concentration of 25 µg/mL were fabricated as previously described [16], modified from the condensation method developed by Sheeran et al. [21]. Briefly, 200 μL of pentobarbital solution in methanol was diluted into ultrapure Milli-Q water to a drug concentration of 1 mg/mL and added to 1.5 mL of Definity lipid solution (0.75 mg/mL lipid concentration). The drug and lipids were combined via tip sonication (S-450D, Branson Ultrasonics; Brookfield, CT, USA) for 20 s of total sonication time, pulsed at 1 s on, 1 s off with a 3 mm tip at 10% power. The solution was crimp-sealed in a 3 mL vial (Wheaton, DWK Life Sciences; Millville, NJ, USA), and the headspace was replaced by DFB gas after thoroughly degassing the vial with a vacuum pump.

To activate, the vial was agitated using a VialMix agitator (Lantheus Medical Imaging; Billerica, MA, USA) for 45 s. The generated precursor microbubbles were condensed by submerging the vial into a bath of isopropanol and dry ice which had been pre-cooled to 0 °C as the vial was being agitated. The vial was swirled gently for 2 min and the bath gradually brought down to −10 °C with additional dry ice. An additional 1 mL of air was injected into the headspace to increase internal vial pressure and facilitate condensation, which is marked by a reduction in visible bubbles and a colour change from an opaque milky white to a more translucent cloudy grey. Free drug and remaining microbubbles were removed by 3 rounds of centrifugation at 300 G for 5 min at 4 °C. The final nanodroplet solution was slowly extruded through a 0.8 μm sterile syringe filter (Minisart Syringe Filter, Sartorius, Göttingen, Germany) to exclude erroneous large droplets. All materials coming into contact with the nanodroplet solution post-activation were kept on ice to limit temperature-related spontaneous vaporization during the fabrication process. Fabricated droplets were stored on ice (approximately 4 °C) and used within 3 h of fabrication or stored at −80 °C for prolonged periods up to 4 weeks. Sham nanodroplets (Sham ND), with no drug loaded, were formulated and stored with the same methods as PBND, minus the drug addition and tip sonication steps.

### 2.3. Physical Characterization

#### 2.3.1. Cryogenic Transmission Electron Microscopy (Cryo-TEM)

ND samples were measured either within 2 h of fabrication or 2 h of thawing from prolonged frozen storage at −80 °C for 2, 7, or 14 days. Images were taken on a Talos L120C TEM (Thermo Fisher Scientific; Waltham, MA, USA) equipped with BM-Ceta metal-oxide semiconductor camera using 120 kV at magnifications 28,000×, 57,000×, and 120,000×. Samples were applied to a grid mesh (Quantifoil R2/2, 300 mesh, Electron Microscopy Sciences), and plunge frozen using a Vitrobot IV (Thermo Fisher Scientific). Transmission electron microscope images were then analyzed with image processing software version 1.53k (ImageJ, National Institutes of Health; Stapleton, NY, USA) to determine nanodroplet diameter and lipid shell thickness. Results were averaged from 3 measurements per imaged droplet.

#### 2.3.2. Size and Nanodroplet Concentration

ND diameter was assessed by dynamic light scattering (DLS; ZetaSizer, Malvern Panalytical, Malvern, Worcestershire, UK) for 3 independent batches at a 1 in 10 dilution of NDs in ultrapure Milli-Q water, and nanoparticle tracking analysis (NTA; NanoSight, Malvern Panalytical, Malvern, Worcestershire, UK) for 2 independent batches, with 3 samples taken from each batch for sample measurements. NTA also yielded a nanodroplet concentration value in particles/mL.

### 2.4. Storage and Stability

#### 2.4.1. Frozen Storage Freeze-Thawing Schedule

After fabrication, PBNDs were aliquoted and frozen at −80 °C. Individual vials were thawed at 4 °C on days 0, 1, 2, 5, 7, 14, and 28 post-frozen storage for characterization. Droplets were used within 15 min of complete thawing.

#### 2.4.2. Stability of Size Distribution and Nanodroplet Concentration

ND size distribution was assessed by DLS for 3 independent batches on all designated post-frozen storage time points (d = 0, 1, 2, 5, 7, 14, 21, 28), in a DTS0012 cuvette. Sizing was corroborated by NTA for 2 independent batches for fresh NDs (d = 0) and 5 independent batches of NDs that had been frozen for 21 days (d = 21). All samples were diluted to 1 in 10 with ultrapure Milli Q water.

#### 2.4.3. Spontaneous Drug Release

To quantify spontaneous drug release in vitro, the thawed nanodroplets were extracted using a hexane organic solvent sink. Equal volumes of the nanodroplets and solvent sink were gently inverted and incubated together on ice for 5 min. Half of the organic solvent sink layer was pipetted into a separate vial for UV-Vis measurements (Cary 6000i UV-Vis-NIR Spectrophotometer; Agilent, Santa Clara, CA, USA).

### 2.5. In Vitro Droplet Activity

#### 2.5.1. In Vitro Acoustic Droplet Vaporization

**Figure 1:**
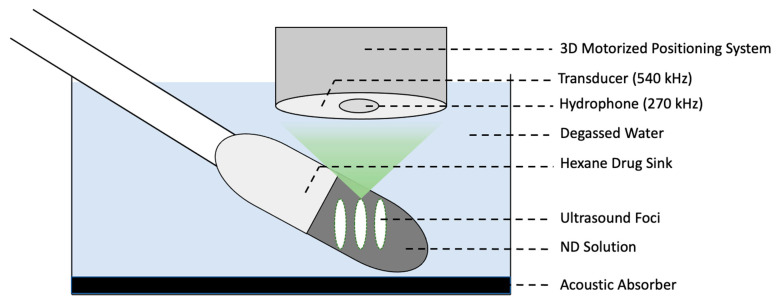
Schematic of in vitro set up to measure ultrasound-triggered droplet vaporization and drug release.

Figure 1 shows a schematic of the in vitro set-up described in Section 2.5. ND aliquots were diluted 1:9 in ultrapure Milli-Q water to a total volume of 2 mL, and were gently pipetted into in a 4 mL polyethylene pipette holder (Millipore Sigma, Massachusetts, USA), which was secured and submerged in warmed degassed water kept at 37 ± 2 °C. Acoustic emissions and drug release from the PBNDs were measured using a prototype preclinical stereotactic-guided focused ultrasound system (RK50, FUS Instruments Inc., Toronto, ON, Canada). FUS-triggered droplet vaporization was assessed by monitoring acoustic emissions resulting from sonicating with a PZT spherically focused transducer with a center frequency of 540 kHz with a 33 mm diameter and a 22 mm radius of curvature, with 10 ms ultrasound pulses at 3 targets, with a pulse repetition frequency of 1 Hz for 120 s. Each independent ND sample was sonicated at one of the following fixed pressures in a randomized order: 0.1, 0.5, 0.9, 1.3, 1.7, 2.0 MPa.

#### 2.5.2. In Vitro Triggered Drug Release

In vitro drug release was performed by adding a 2 mL organic solvent sink layer of hexane on top of the ND sample as described in Section 2.4.3 in the 4 mL volume polyethylene pipette. Hexane was added immediately before sonication, and a rubber stopper was used to seal the holder to prevent evaporation. The ultrasound focus was contained within the ND layer. Following sonication, the bulb of the pipette was gently squeezed 10 times and the polyethylene pipette was left on ice for drug dissolution testing for 4 min. A 1 mL sample of the organic layer was extracted and stored in PTFE-lined nonreactive glass vials (Millipore Sigma, Massachusetts, USA). The total elapsed time between hexane addition and extraction was approximately 7.5 min. The amount of ultrasound-triggered pentobarbital released was quantified by measuring UV-Vis absorbance (Cary UV-Vis-NIR Spectrophotometer, Agilent, Santa Clara, CA, USA), subtracting absorbance values of sham (no-drug loaded) NDs, and comparing to a standard curve of the drug prepared in various concentrations in hexane.

### 2.6. Hemolysis Assay

Intravenous whole blood was collected from rodents in BD Vacutainer™ sodium citrate blood collection tubes (Thermo Fisher Scientific, USA) and washed 3 times at 500 G for 10 min with saline (NaCl 0.9%), aspirating after every wash. Red blood cells (RBCs) were resuspended in saline and stored at 4 °C for future use. Blood samples were all used with 1 week of collection. Before use, RBCs were isolated by centrifugal spinning at 500 G, and aliquots of 250 µL RBCs were incubated in the following conditions: saline (4 °C and 37 °C), sham NDs (1×, 5×, and 10×), PBNDs (1×, 5×, and 10×), and Triton X-100 (4 °C and 37 °C). Nanodroplets at 1×, 5×, and 10× refer to equivalent concentrations in 25% hematocrit blood, which would correspond to multiples of 1.5 mL/kg of NDs in vivo. The nanodroplet concentrations were chosen to represent the dosage equivalents used in previous studies [15,16] sufficient to achieve a significant therapeutic effect. All nanodroplets were incubated at 37 °C to mimic in vivo conditions, for 30, 60, or 120 min which is in excess of the 8 min ND circulation half-life in vivo previously measured [15]. Triton X-100 and PBS were used as positive and negative controls, respectively. Lysis was assessed via separation of lysed cells and intact RBCs by centrifugation at 500 G for 10 min and the absorbance (Synergy H1, Biotek; Winooski, VT, USA) of the supernatant at 540 nm was measured. All incubation conditions were repeated 3 times with independent ND and blood samples. Sample brightfield microscopy images (Zeiss Axio Observer Z1; Carl Zeiss, Oberkochen, Germany) were taken at 63× magnification to observe healthy RBCs (round, dimple shape) or lysed cells (swollen, lost shape, disintegrated cell wall).

### 2.7. Statistical Analysis

Single-factor analysis of variance (ANOVA) was performed for the frozen ND diameter, polydispersity index of distribution, drug release, and acoustic emissions, as well as hemolysis incubation groups. Significant differences were identified using a post hoc *t*-test for a *p*-value < 0.05. Linear regression analysis using a generalized linear model was used to determine whether correlation existed between shell thickness and ND diameter from cryo-TEM images, and between drug release and acoustic emissions for frozen samples, with a cut-off set at 0.7 for the multiple R^2^ value and *p*-value < 0.05.

## 3. Results

### 3.1. Physical Characterization

#### 3.1.1. Cryo-TEM

**Figure 2:**
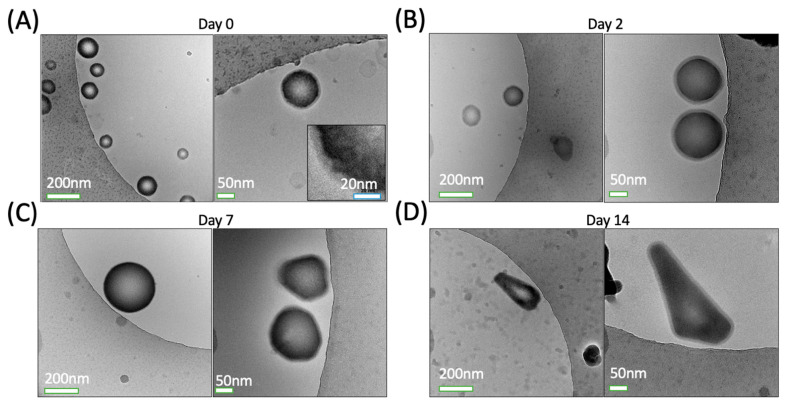
Sample cryo-TEM images of volatile perfluorocarbon NDs (**A**) fresh, and frozen at −80 °C for (**B**) 2, (**C**) 7, and (**D**) 14 days, respectively. Scale bars represent 200 nm and 50 nm, respectively for left (28,000× magnification) and right (57,000× magnification) images in each set, and 20 nm for the inset (120,000× magnification) in (**A**).

**Figure 3:**
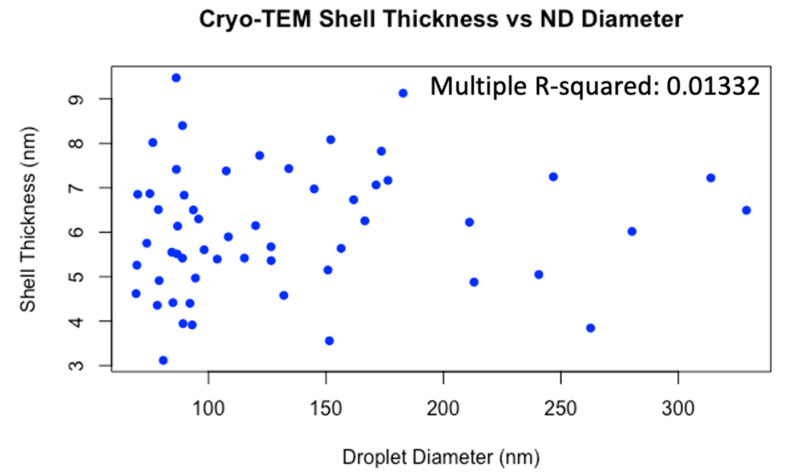
Nanodroplet lipid shell thickness plotted against diameter from cryo-TEM image analysis.

Figure 2 shows example cryo-TEM images of fresh NDs (Figure 2A) and thawed NDs post-freezing (Figure 2B–D). NDs typically appeared spherical with a textured thick shell surrounding the electron-dense perfluorocarbon core, and smooth outer edge between the lipid shell and surrounding medium. Image analysis yielded an average ND diameter of 132 ± 64.4 nm, and lipid shell thickness of 6.0 ± 1.4 nm (average ± standard deviation). To our knowledge, this is the first published report of volatile perfluorocarbon NDs imaged with cryo-TEM, providing a quantification of the lipid shell thickness. ND diameter was found to be independent of lipid shell thickness, based on the low multiple R^2^ = 0.0133 from a linear regression model, which indicates droplet diameter is a poor predictor of shell thickness (Figure 3), with a *p*-value of 0.401 indicating a low chance of statistically significant linear relation between the two factors.

#### 3.1.2. Size and Concentration

**Figure 4:**
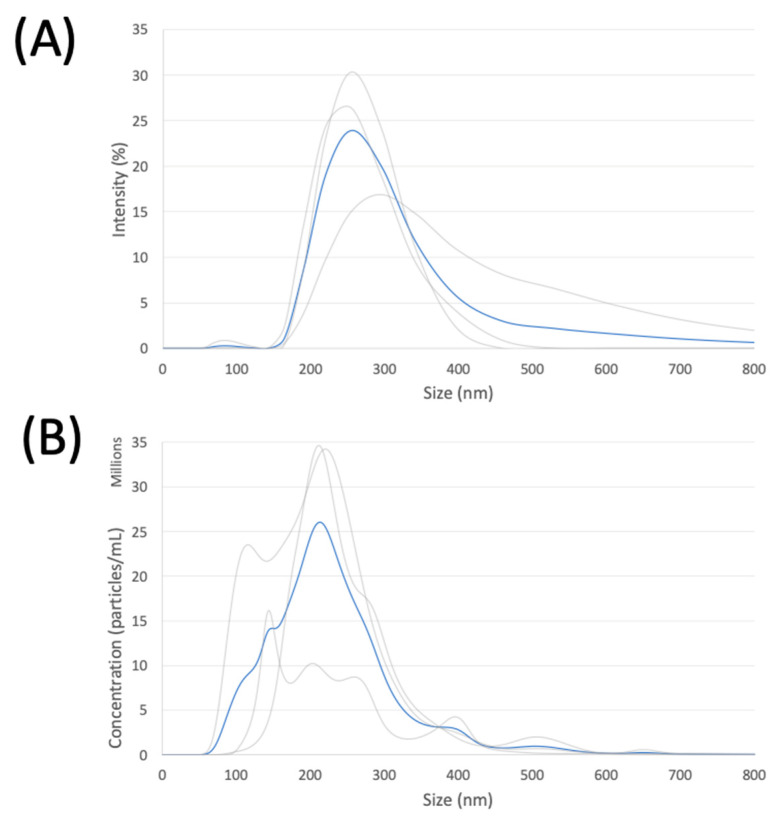
Nanodroplet hydrodynamic diameter as measured by (**A**) dynamic light scattering, and by (**B**) nanoparticle tracking analysis, for (n = 3) independently fabricated batches of fresh PBNDs, within 3 h of fabrication for each measurement method. Measurements of individual batches are plotted in gray, and the mean of those measurements is plotted in blue.

NDs measured via dynamic light scattering were 329 ± 27.6 nm and 237 ± 3.3 nm via nanoparticle tracking analysis, respectively, for fresh nanodroplets within 3 h of fabrication and kept on ice (Figure 4). The average concentration of fresh droplets was measured to be 4.08 × 10^9^ ± 8.55 × 10^4^ droplets/mL, which is 2.46 times higher than those of frozen droplets.

### 3.2. Storage and Stability

#### 3.2.1. Cryo-TEM

ND morphology changed with prolonged storage at −80 °C. Droplets captured via cryo-TEM showed a loss in circularity and a greater number of free liposomes as the number of days frozen increased (Figure 2). Additionally, fewer droplets were present in the samples.

#### 3.2.2. Size and Concentration

**Figure 5:**
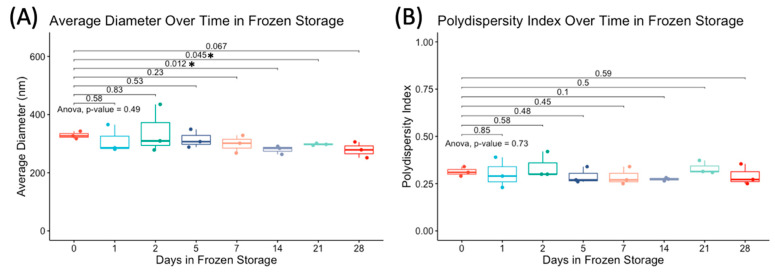
Change in (**A**) average diameter and (**B**) PDI of n = 3 independent batches of nanodroplets fabricated on separate days over time in frozen storage at −80 °C. The *p*-values of an ANOVA between fresh droplets and each subsequent day are displayed above the box plots. Asterisk (*) used to denote statistically significant (*p* < 0.05) results.

The average and standard deviation of hydrodynamic diameter and polydispersity index (PDI) of fresh and frozen NDs at days 0, 1, 2, 5, 7, 14, 21, and 28 are given in Table 1. Each average is a result of three DLS measurements from three independently fabricated batches of droplets. As shown in Figure 5, an analysis of variance across the days frozen showed the diameter to be significantly (*p* < 0.05) smaller for days 14 and 21 compared to fresh droplets. However, there was no significance for droplets frozen less than 2 weeks when compared to fresh droplets.

NTA measurements to compare droplets that had been frozen at −80 °C for 21 days and NDs that were freshly fabricated corroborate the DLS measurements showing that the mean ND diameter remains unchanged after frozen storage. As shown in Table 2, there was a significant decrease in ND concentration measured by NTA after 21 days of frozen storage, potentially due to the largest fabricated droplets being more susceptible to spontaneous vaporization during the freeze–thaw process.

### 3.3. Droplet Activity

#### 3.3.1. Acoustic Droplet Vaporization

**Figure 6:**
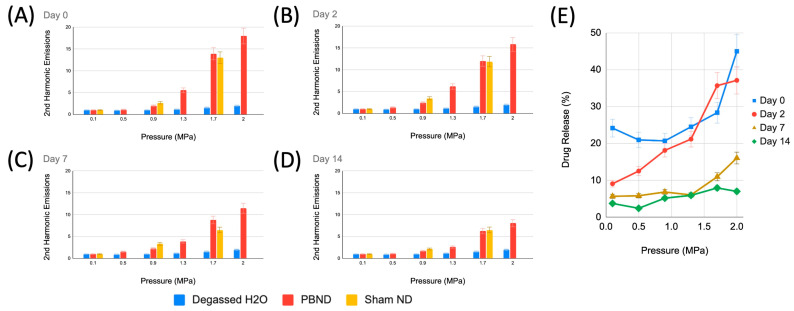
Graphs depicting the magnitude of the second harmonic emission from vaporized pentobarbital-loaded nanodroplets (PBNDs) and sham nanodroplets (sham NDs) as a result of focused ultrasound sonication with a 540 kHz transducer at various fixed pressures, for droplets frozen at −80 °C for (**A**) 0, (**B**) 2, (**C**) 7, and (**D**) 14 days, respectively. Arbitrary units. (**E**) Released drug at each sonication pressure are shown as a percentage of the total drug loaded in fresh and frozen PBNDs. Error bars represent one standard deviation.

**Figure 7:**
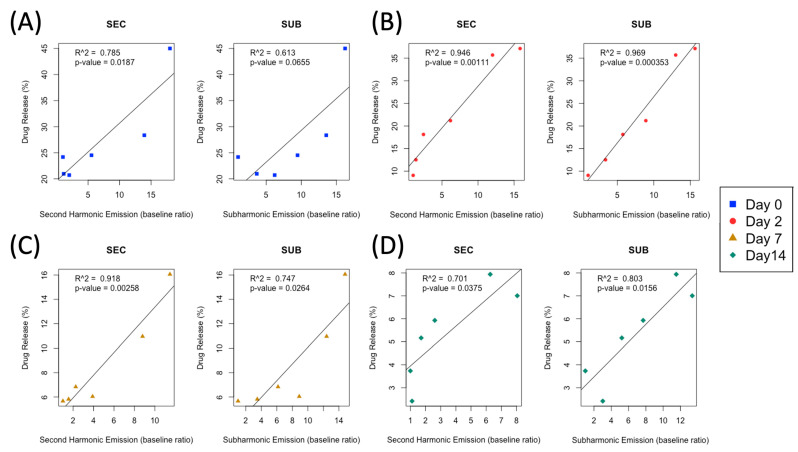
Linear regression analysis of drug release and acoustic emissions at the second- and sub-harmonic frequencies, triggered with a 540 kHz transducer for (**A**) fresh PBNDs and frozen PBNDs for (**B**) 2 days, (**C**) 7 days, and (**D**) 14 days. Metrics for linear regression analysis, the *p*-value, and multiple R^2^ values for each set of analysis are shown. *p*-value represents the probability that there is no predictive correlation between acoustic emissions and drug release, where a smaller value represents a higher chance of a relationship between the two factors. Multiple R^2^ value is a measure of goodness of fit of the data to a linear regression model.

The magnitude of the second harmonic and subharmonic frequency emissions were used as indicators of acoustic droplet vaporization. The magnitude of the second harmonic emission was found to be a predictor of drug release, whereby the magnitude of the second harmonic emission correlated with the quantity of drug released (Figure 6 and Figure 7). Fresh droplets (Figure 6A) showed an increase in the second harmonic signal from baseline of more than 15 times, at a sonication pressure of 2.0 MPa. There was no statistically significant change in the second harmonic emission produced by fresh and frozen droplets after 2 days (Figure 6B). However, there was a significant reduction in acoustic emissions after 7 and 14 days of frozen storage (Figure 6C,D). Sham NDs exhibited the same trends and exhibited similar levels of acoustic emissions with respect to PBNDs. Degassed water, the negative control case, in the same set-up and experimental conditions, typically saw lower levels of acoustic emissions in comparison to both PBNDs and Sham NDs, particularly at sonication pressures higher than 0.9 MPa, where the differences were significant.

#### 3.3.2. Pressure-Dependent, Ultrasound-Triggered Drug Release

Drug release, as determined by the absorbance measurements of the organic solvent sink, is expressed as a percentage of the total amount of drug released from spontaneous vaporization of PBNDs at room temperature over 3 h. At 2.0 MPa sonication pressure, PBNDs achieved 45%, 37%, 16%, and 7% drug release when frozen for 0 (fresh), 2, 7, and 14 days, respectively (Figure 6E). A linear regression analysis between absorbance measurements and acoustic emissions at the second harmonic frequency (the best predictor out of the examined frequencies), was applied to each set of frozen droplets (i.e., day 0, 2, 7, 14), the r-squared values were fresh = 0.79, day 2 = 0.95, day 7 = 0.92, and day 14 = 0.70 (Figure 7), all of which are above the threshold of 0.7, indicating a correlation between acoustic response and drug release. Acoustic emissions are quantified as a baseline ratio, meaning the ratio of the harmonic peak magnitude with and without PBNDs present.

### 3.4. Hemolysis Assay

**Figure 8:**
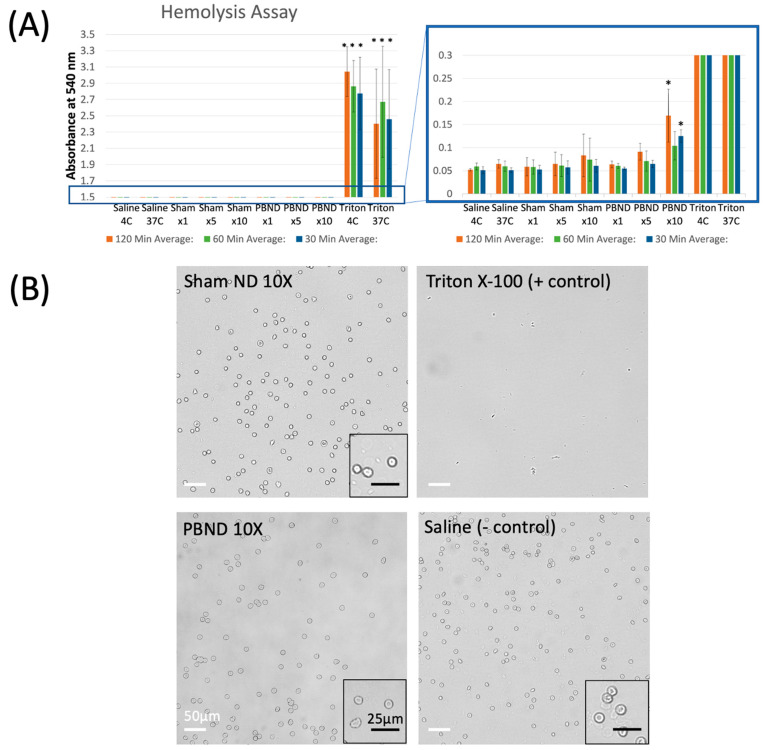
Results of the hemolysis assay. (**A**) Absorbance measurements at 540 nm of released hemoglobin from lysed red blood cells incubated in various conditions. Asterisks represent statistically significant differences from the negative control case, saline at 4 °C. (**B**) Sample brightfield images from confocal microscopy of RBC samples post-incubation in various solutions for 60 min at 37 °C. Scale bar (white): 50 µm; scale bar (black): 25 µm. “*” = *p* < 0.05; “***” = *p* < 0.001.

Incubating PBNDs or sham NDs with RBCs produced no significant hemolysis at therapeutic concentrations. No observable difference in absorbance (a measure of hemolysis) was found between incubation at 4 °C and 37 °C for the positive and negative controls. Absorbance levels for all sham ND concentrations, 1X-PBND, and 5X-PBND at all tested incubation times were not statistically different from saline, the negative control (Figure 8A). Triton X-100 (positive control condition), 10X-PBND at 30 and 120 min did show a significant rise in absorbance in comparison to saline.

The sample microscopy images shown in Figure 8B show the morphology of RBCs incubated with saline, 10X-sham ND, 10X-PBND, and Triton X-100 at 37 °C. All images were taken at the same magnification and dilution, and the 10X-sham ND images were qualitatively the most similar to the negative control condition. In contrast, the samples incubated with 10X-PBND appeared to have a lower concentration of viable RBCs. The positive control incubation with Triton X-100 resulted in no RBCs remaining. The results of confocal microscopy appear to match the results of the hemolysis assay.

## 4. Discussion

**Figure 9:**
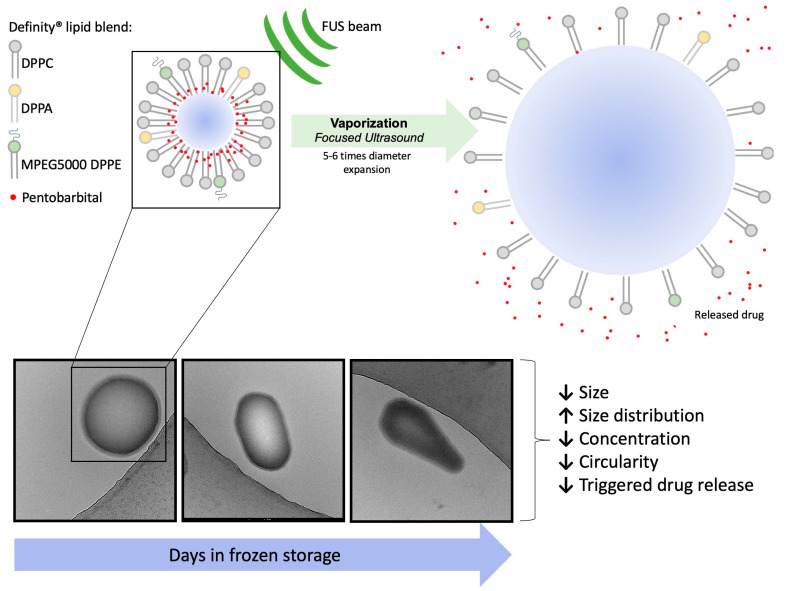
Schematic showing the drug-loaded Definity-based nanodroplet-releasing drug in response to focused ultrasound, and main findings of investigation of frozen storage.

Here, we show the fabrication of anesthetic-loaded nanodroplets by repurposing Definity microbubbles, achieving a consistent size distribution across multiple batches, measured with DLS, NTA, and cryo-TEM. Frozen storage was shown to be an effective method to preserve size, drug loading, and triggered-release over 7 days stored at −80 °C. Incubation of PBNDs with RBCs at above therapeutic concentrations showed no signs of hemolysis (Figure 9, Table 3).

### 4.1. Droplet Sizing

Nanotherapeutic effectiveness is highly dependent on physical characteristics such as nanoparticle size, shape, and surface chemistry [22]. In particular, nanoparticle size has a profound effect on in vivo pharmacokinetics, including biodistribution, circulation half-life, and cellular uptake, and therefore, the overall effectiveness and feasibility of the treatment itself [23]. Consistent size distributions of ultrasound-activated nanodroplets are important in determining the ultrasound parameters required for optimal drug release and safety, as nanodroplet size affects the acoustic vaporization threshold and cavitation activity [13].

It may be of interest to note that there is a size difference of around 100 nm between DLS and NTA, with DLS reporting larger. This difference can be attributed to the fact that neither obtain a ‘direct measurement’ of particle diameter, and instead, dynamic light scattering and nanoparticle tracking analysis measure the hydrodynamic diameter, which is inevitably larger than the true diameter. The scattering intensity fluctuations as recorded from dynamic light scattering may be more sensitive to skewing by larger outliers. The average ND diameter measured via cryo-EM is smaller than both those using DLS and NTA, likely due to the fact that larger droplets are more likely to be blotted away to achieve a single layer of droplets during the cryogenic freezing process. Since only a small population of NDs was measured via cryo-EM to gain insight to ND morphology, circularity, and whether there is any correlation between shell thickness and shell diameter, the center of the diameter distribution for the ND population is likely between the 329 ± 28 nm and 237 ± 3 nm measured via DLS and NTA, respectively.

Although there is a slight difference in the measured nanoparticle diameter across quantification methods, we are able to consistently trigger similar degrees of vaporization and drug release, indicating our fabricated droplets do not have vastly differing size distributions across samples (since large size deviations would have a significant impact on ultrasound-triggered activity).

### 4.2. Droplet Morphology–Cryo-TEM

Since most nanomedicines are <300 nm in diameter [7] (including FUS-activated nanodroplets which average ~200 nm)—sizes smaller than the wavelengths of visible light—light-based microscopy cannot accurately determine nanoparticle morphology. Instead, electron microscopy (EM) techniques such as transmission electron microscopy, scanning electron microscopy, and cryo-EM are examples of techniques that allow for high-resolution imaging including nanoparticles [24].

Cryo-electron microscopy has been widely applied to analyze liposomal structures [25], including acoustically active echogenic structures such as liposomes [26] and microbubbles [27,28], but not for low-boiling-point nanodroplets. Furthermore, previous reports examine the phospholipid bilayer of their liposomal structures by uranyl acetate staining. Cryo-TEM images of NDs show a clear distinction between perfluorocarbon core, lipid shell, and surrounding media, thus allowing for the development of a method of direct measurement of shell thickness without staining.

### 4.3. Droplet Stability–Frozen Storage Potential

Frozen storage was assessed to evaluate the possibility of nanodroplets being fabricated in advance and stored at −80 °C for up to 4 weeks before being thawed and used for theranostic purposes in vivo. Advance fabrication capability is an attractive trait, as the delivery system’s usage would be less demanding on researchers and/or medical professionals in a clinical setting. As can be observed in Figure 5, there is a marginal effect of frozen storage times on nanodroplet size and consistently low values for polydispersity index (PDI) were measured, which indicates drug-loaded droplets can be fabricated in advance and kept in frozen storage. However, droplet concentration (Table 2) and corresponding drug release (Figure 6) decreased significantly as frozen storage period increased. Cryo-TEM images (Figure 2) of the PBND samples support a hypothesis that the perfluorocarbon partially diffused out of the spherical NDs, leaving behind irregular-shaped droplets and lipid fragments which retain drug, but are not acoustically active. Thus, nanodroplet concentration and drug dosages should be calculated prior to frozen storage. To improve thermal stability, a possible solution to consider in the future is layer-by-layer assembly of perfluorocarbon nanodroplets [29].

### 4.4. Efficacy Assessment

In general, a sigmoidal curve would be expected to form between second harmonic emissions and pressure as nanodroplets are vaporized to microbubbles, plateauing when the population of nanodroplets is depleted [6]. However, in our preclinical FUS system, a pressure cut-off for each transducer frequency is made considering the mechanical index and other safety measures. Therefore, we only see the exponentially rising phase of the curve. Acoustic emissions higher than negative control indicate ultrasound-induced vaporization of our NDs can be acoustically monitored, and similarity between sham NDs and pentobarbital-loaded NDs suggests that the presence of drug does not significantly affect nanodroplet acoustic behavior or ability to be vaporized.

In vitro, a decreasing trend in relative acoustic emissions as NDs are frozen for longer periods of time indicates that fewer viable NDs are present in the sample. This hypothesis is supported by the decrease in sample concentration (Table 2), and cryo-TEM images (Figure 2) which show fewer NDs and more lipid fragments, likely a consequence of the decafluorobutane dissolution.

Furthermore, the decreasing percentage of drug release matches with the decreased acoustic emissions as PBNDs are frozen for longer time periods. Similar to the acoustic emissions, as the pressure increases, the amount of absorbance, and therefore drug released, also increases, evidencing a pressure-dependent drug release. Compared to the negative control of sham NDs, the absorbance levels in the organic drug sink were consistently higher in PBND cases, with the disparity becoming particularly evident as sonication pressures were increased.

To ascertain this relationship, a linear regression analysis was performed between second harmonic acoustic emissions and absorbance (Figure 7). Other than PBNDs frozen for 2 days, the remaining droplet samples showed that as the number of days frozen increases, the slope of absorbance as a function of acoustic emissions decreases. In summary, this indicates that droplets frozen for a longer period released a smaller amount of drug, even with the same FUS stimulation parameters. Finally, the *p*-values associated with this regression model were all <0.05, indicating a high confidence in acoustic emissions being predictive of drug release.

### 4.5. Cytotoxicity–Hemolysis Assay

After incubating PBNDs with RBCs, no significant cell lysis was observed in conditions up to 5 times the effective in vivo treatment dose, for incubation periods up to 120 min, which is much longer than the nanodroplet circulation half-life [15]. This suggests that no significant RBC lysis would be observed with our current treatment schemes. Incubation conditions at concentrations higher than and at intervals longer than treatment serve to provide a sense of maximum tolerated dosages and serve as a guideline for future in vivo studies should it become desirable to increase treatment dosage.

## 5. Conclusions

In conclusion, through thorough characterization, we have shown that volatile perfluorocarbon NDs are a powerful vehicle for US-mediated drug release, a process which can be monitored and controlled via acoustic emissions both in vitro and in vivo [30]. We evaluated whether NDs could be stored at −80 °C for prolonged periods to lessen the logistical burden of ND fabrication. The results show that the drug-loaded NDs can be stored for up to 1 week until they begin to lose their structure (due to perfluorocarbon diffusing out of droplets), and the lipophilic drug becomes trapped in smaller lipid fragments or liposomes, causing a reduction in acoustic activity and drug release. We have demonstrated for the first time that volatile NDs can be imaged with cryo-TEM (not possible with conventional TEM), which gives insights about their internal composition, electron density, and shell thickness. Finally, PBNDs result in no adverse interactions when incubated with RBCs at concentrations exceeding what is therapeutically effective.

## Figures and Tables

**Table 1:** Measured nanodroplet diameter and polydispersity index (PDI) from three independent batches of nanodroplets fabricated on separate days.

Days Frozen	Average Diameter (nm)	Diameter St. Dev (nm)	Average PDI	PDI St. Dev
0	329.00	27.56	0.32	0.04
1	310.79	27.65	0.30	0.04
2	341.01	67.74	0.34	0.08
5	314.90	15.49	0.29	0.08
7	299.34	13.01	0.29	0.03
14	279.49	7.13	0.27	0.04
21	297.88	16.61	0.33	0.03
28	278.80	13.59	0.29	0.03

**Table 2:** Measurements of nanodroplet diameter mean, mode, standard deviation, and concentration (± standard error values) as measured by nanoparticle tracking analysis.

Days Frozen	Mean Diameter (nm)	Mode Diameter (nm)	St. Dev (nm)	Concentration (Particles/mL)
0	237 ± 3.3	192 ± 7.9	96 ± 3.7	4.08 × 10^9^ ± 8.55 × 10^4^
21	250 ± 3.4	206 ± 7.9	90 ± 1.6	1.66× 10^9^ ± 3.02 × 10^4^

**Table 3:** Summary of main findings of the investigation.

	Primary Outcome(s)	Main Finding(s)
Dynamic Light Scattering	ND hydrodynamic diameter Polydispersity of size distribution	Mean hydrodynamic diameter significantly different after 14 days frozen Monodisperse size up to 14 days frozen
Cryo-EM	Nanodroplet morphology Lipid shell thickness	As time frozen increases, NDs lose circularity. Shell thickness unrelated to ND size
Nanoparticle Tracking Analysis	ND hydrodynamic diameter Particle concentration	ND concentration decreased after 14 days frozen
Acoustic activity	Subharmonic and second harmonic acoustic emissions	Magnitude of harmonic emissions decreased with prolonged frozen storage
Drug release	Drug release percentage	Drug release percentage decreases as time frozen increases
Focused ultrasound- triggered drug release	Subharmonic and second harmonic acoustic emissions Drug release percentage	Correlation found between acoustic response and drug release, indicating pressure dependent-drug release possible with NDs.
Hemolysis Assay	Absorbance from hemolysis	No significant hemolysis of RBCs detected after incubation with NDs up to 10 times the therapeutic dose

## Data Availability

The data presented in this study are available from the corresponding author upon reasonable request.
